# Combined Use of Zoledronic Acid Augments Ursolic Acid-Induced Apoptosis in Human Osteosarcoma Cells through Enhanced Oxidative Stress and Autophagy

**DOI:** 10.3390/molecules21121640

**Published:** 2016-11-30

**Authors:** Chia-Chieh Wu, Yi-Fu Huang, Chen-Pu Hsieh, Pin-Ju Chueh, Yao-Li Chen

**Affiliations:** 1 Orthopedics & Sports Medicine Laboratory, Changhua Christian Hospital, Changhua 50006, Taiwan; 50560@cch.org.tw (C.-C.W.); 181064@cch.org.tw (Y.-F.H.); 51114@cch.org.tw (C.-P.H.); 2 Department of Orthopedic Surgery, Changhua Christian Hospital, Changhua 50006, Taiwan; 3 Institute of Biomedical Sciences, National Chung Hsing University, 145 Xingda Rd., South Dist., Taichung 40227, Taiwan; 4 School of Medicine, Kaohsiung Medical University, Kaohsiung 80708, Taiwan; 5 Department of Biotechnology, Asia University, Taichung 41354, Taiwan; 6 Graduate Institute of Basic Medicine, China Medical University, Taichung 40402, Taiwan; 7 Department of Medical Research, China Medical University Hospital, Taichung 40402, Taiwan; 8 Transplant Medicine & Surgery Research Centre, Changhua Christian Hospital, Changhua 50006, Taiwan; 9 Department of Surgery, Changhua Christian Hospital, 135 Nansiao St., Changhua 50006, Taiwan

**Keywords:** zoledronic acid, ursolic acid, osteosarcoma

## Abstract

Ursolic acid (UA), a naturally occurring pentacyclic triterpene acid found in many medicinal herbs and edible plants, triggers apoptosis in several tumor cell lines but not in human bone cancer cells. Most recently, we have demonstrated that UA exposure reduces the viability of human osteosarcoma MG-63 cells through enhanced oxidative stress and apoptosis. Interestingly, an inhibitor of osteoclast-mediated bone resorption, zoledronic acid (ZOL), also a third-generation nitrogen-containing bisphosphonate, is effective in the treatment of bone metastases in patients with various solid tumors. In this present study, we found that UA combined with ZOL to significantly suppress cell viability, colony formation, and induce apoptosis in two lines of human osteosarcoma cells. The pre-treatment of the antioxidant had reversed the oxidative stress and cell viability inhibition in the combined treatment, indicating that oxidative stress is important in the combined anti-tumor effects. Moreover, we demonstrated that ZOL combined with UA significantly induced autophagy and co-administration of autophagy inhibitor reduces the growth inhibitory effect of combined treatment. Collectively, these data shed light on the pathways involved in the combined effects of ZOL and UA that might serve as a potential therapy against osteosarcoma.

## 1. Introduction

Osteosarcoma is also known as osteogenic sarcoma which arises from osteoid tissue in the bone. This type of tumor most often localizes in the metaphysis of the adolescent long bones [1], which is characterized by a high propensity for metastasis, especially in lung. The outcome for this metastatic potential is frequently associated with high incidence of death in patients [2]. The current treatments for osteosarcoma include surgery, radiation therapy, chemotherapy and other new forms of treatments, such as immunotherapy and targeted therapy. Unfortunately, there is a lack of effective cures for most patients suffering from advanced osteosarcoma.

Recent progress has focused on the chemoprevention by natural products for their anti-growth activity against cancer cells and may exhibit less side effects compared to synthetic compounds. Ursolic Acid (UA) (3β-hydroxy-urs-12-en-28-oic acid) is a pentacyclic triterpenoid compound found in apple peels, and in the Ayurveda herb known as Holy Basil. UA is shown to trigger apoptosis, leading to anti-cancer, anti-invasion, anti-metastasis, anti-proliferation and anti-angiogenesis in an array of human cancer lines [3,4,5,6,7,8]. However, the studies on the effect of UA on human bone cancer cells are fairly limited. In this regard, we have just reported that UA also suppresses human osteosarcoma MG-63 cell growth through enhanced oxidative stress and ERK1/2-MAPK-assosiated apoptotic pathway [9].

Zoledronic acid (ZOL), a third-generation nitrogen-containing bisphosphonate, is an inhibitor of osteoclast-mediated bone resorption and has demonstrated its efficacy in the treatment of bone metastases in cancer patients with breast [10,11,12,13], prostate [14,15,16], lung, and other solid tumors [17]. Data in in vitro studies also support that ZOL inhibits osteosarcoma cell growth through activation of immune system, suppression of angiogenesis and apoptosis induction [18,19,20]. Interestingly, the use of ZOL has also been shown to reverse drug resistance in osteosarcoma [21]. Moreover, ZOL has the ability to reduce primary tumor growth, decrease lung metastases and prolong survival in animal models of osteosarcoma [22,23,24]. In a four-patient cohort study, following initiation treatment of ZOL with high-grade osteosarcoma, the median progression-free survival was increased to 19 months, and median overall survival was increased to longer than 56 months [25]. Given that ZOL exhibits great potential as an anti-cancer agent in bone cancer cells, we therefore sought to study the additive effect of the combination of ZOL and UA on human bone cancer cells.

## 2. Results

**Figure 1:**
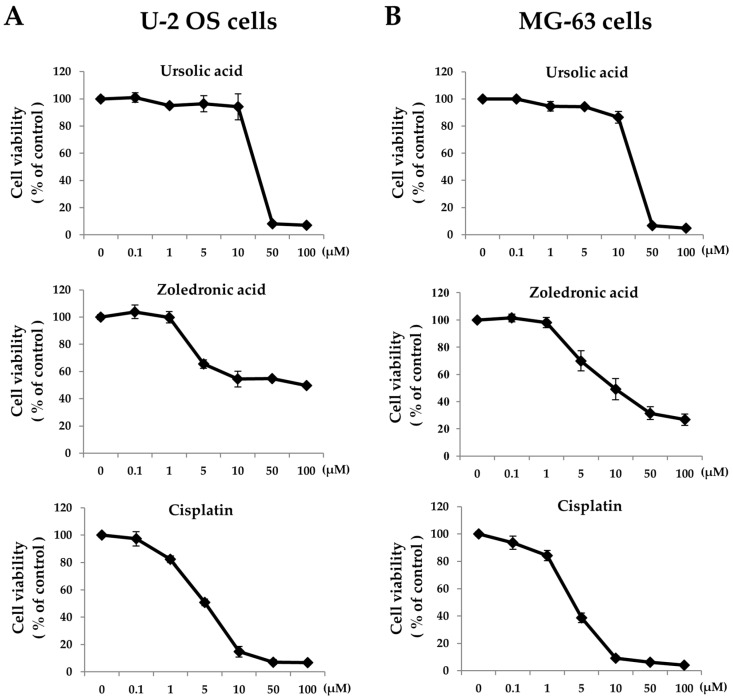
Zoledronic acid, ursolic acid, and cisplatin differentially affect osteosarcoma cell viability. MTT assays were performed with U-2 OS cells (**A**); or MG-63 cells (**B**) treated with either Zoledronic acid (ZOL), ursolic acid (UA), or cisplatin (CPT) at the indicated concentrations for 72 h. Three independent experiments were conducted.

**Figure 2:**
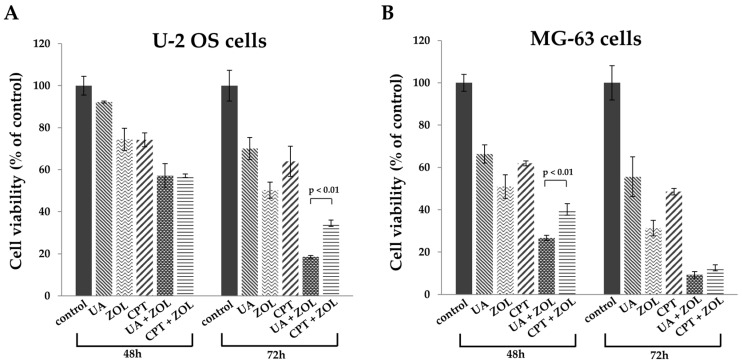
Zoledronic acid enhances ursolic acid-mediated inhibition of cell viability. MTT assays were performed with U-2 OS cells (**A**) or MG-63 cells; (**B**) treated with either ZOL (10 μM), UA (20 μM), CPT (5 μM) or a combination of ZOL/UA, or ZOL/CPT at different time points. Three independent experiments were conducted.

Zoledronic acid and ursolic acid, alone or in combination, reduced osteosarcoma cell viability and proliferation. Cell viability was examined using MTT (3-(4,5-dimethylthiazol-2-y1)-2,5-diphenyltetrazolium bromide) assay and we found that UA significantly reduced cell viability at 50 μM on both U-2 OS and MG-63 cells. Similar results were also observed in cells exposed to ZOL, where significant inhibition was reached with concentrations ranging from 5 to 100 μM (Figure 1). The half-maximal inhibitory concentration (IC_50_) calculated based on data from the MTT assays for U-2 OS cells were 28.3 μM (UA) and 50.7 μM (ZOL) and those for MG-63 were 27.2 μM (UA) and 40.4 μM (ZOL). Cisplatin (CPT) is a clinical anti-cancer compound and we showed that its order of effectiveness was similar to that observed for UA and ZOL (Figure 1). Subsequently, we studied cell viability in two lines of bone cancer cells exposed to either single compound, or UA/ZOL and CPT/ZOL in combination. We observed significant anti-growth effect with UA/ZOL, and CPT/ZOL combination in both U-2 OS and MG-63 cells. In order to study the combinational effect of these compounds, we decided to use 20 μM for UA and 10 μM for ZOL. Interestingly, the combined effect of UA and ZOL was stronger compared to the combined use of CPT and ZOL in U-2 OS cells at 72 h (Figure 2A) and in MG-63 cells at 48 h (Figure 2B). These results clearly demonstrate that UA and ZOL, alone or in combination, significantly reduce osteosarcoma cell viability.

**Figure 3:**
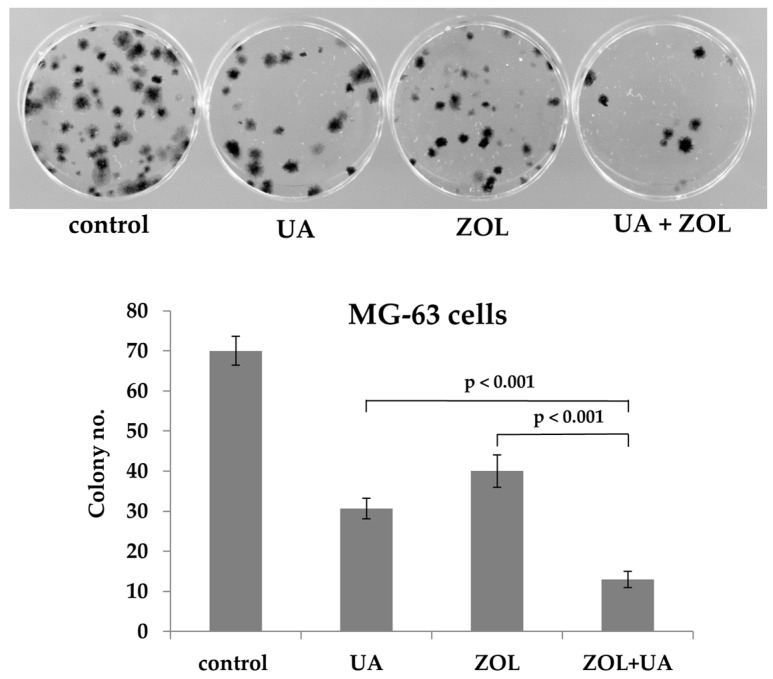
Zoledronic acid enhances ursolic acid-mediated reduction of colony formation. MG-63 cells were plated in colony formation assays after treatment with either ZOL (10 μM), UA (20 μM), or a combination of ZOL/UA for 6 h. Five hundred cells were plated per dish. All experiments were performed in triplicate, and the figure above shows a representative example.

We next examined the combined effect of UA and ZOL on cell proliferation capacity (also the inhibitory effect of these compounds on cell growth) by colony formation assays. Consistent with the data from MTT assays, the combined effects of UA and ZOL were further confirmed by the greatly reduced colony number compared to that of control or single-compound treatment in MG-63 cells (Figure 3). These two lines of evidence strongly support that UA and ZOL, alone or in combination, markedly attenuate the growth of bone cancer cells.

### 2.1. Zoledronic Acid and Ursolic Acid, Alone or in Combination, Mediated Apoptosis in Osteosarcoma Cells

**Figure 4:**
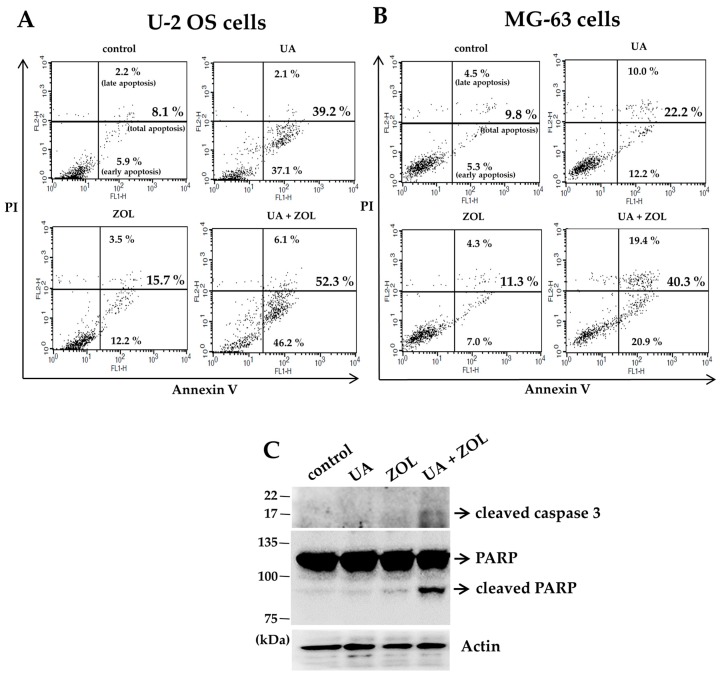
Zoledronic acid combines with ursolic acid to enhance apoptosis. U-2 OS cells (**A**); or MG-63 cells (**B**) were treated with either ZOL (10 μM), UA (20 μM) or a combination of ZOL and UA for 48 h. To detect apoptosis, the cells were stained with Annexin-V and propidium iodide (PI), and analyzed using flow cytometry. Numbers indicated the percentage of Annexin-V positive cells; Expression of apoptosis related proteins was measured by Western blotting in MG-63 cells (**C**).

To determine whether programmed cell death is involved in the combined anti-proliferative effect of UA/ZOL, we analyzed cells for apoptotic subpopulations. In both lines of U-2 OS and MG-63 cells, a 48-h exposure of either single compound or in combination all induced a significant increase in apoptosis (Figure 4A,B). More importantly, cells exposed to combinational treatment exhibited enhanced apoptosis compared with those of single compound treatment, supported by the enhanced activated caspase-directed poly(ADP-ribose) polymerase (PARP) cleavage (Figure 4C).

**Figure 5:**
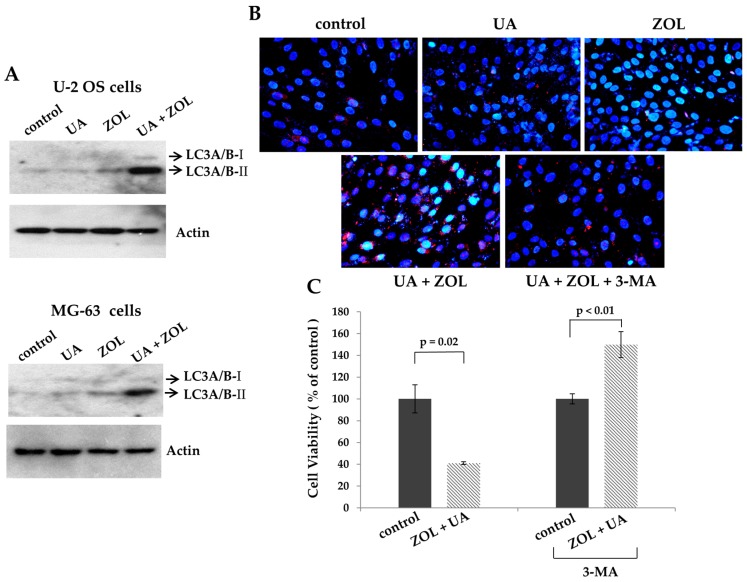
Autophagy is involved in the anti-proliferative activity of zoledronic acid/ursolic acid combination. (**A**) zoledronic acid combines with ursolic acid to induce the expression of LC3A/B-II. U-2 OS cells or MG-63 cells were treated with either ZOL (10 μM), UA (20 μM) or a combination of ZOL and UA for 48 h. Cells were harvested and the cell lysates were analyzed by Western blotting using indicated antibodies; (**B**) zoledronic acid combines with ursolic acid to induce acidic autophagic vacuoles. MG-63 cells were treated with either ZOL (10 μM), UA (20 μM) or a combination of ZOL and UA in the absence or present of 3-MA (2 μM) for 36 h. The cells were stained with acridine orange (AO) to observe acidic (autophagic) vacuoles (**red** color), and 4',6-diamidino-2-phenylindole (DAPI) for labeling nucleus (**blue** color); and (**C**) autophagy inhibitor blocks the anti-proliferative effect caused by the UA/ZOL combination. U-2 OS cells were treated with ZOL (10 μM) and UA (20 μM) in the absence or presence of 3-MA (2 μM) for 48 h. The treated cells were processed to MTT assays to measure cell viability. Cell viability of drug-treated cells was compared to their own control cells in the absence or presence of 3-MA.

We also investigated the probability that these compounds induce autophagy-related mechanism, a type II programmed cell death, in these cells. Autophagy is a self-digestion and bulk-degradation mechanism that protects cells in response to stresses; on the other hand, it may be cytotoxic depending on cell type and their context. Protein analysis has revealed that the ZOL exposure increased the formation of LC3A/B-II, an indication for autophagy induction, compared to that of control or UA exposure (Figure 5A). Interestingly, the expression of LC3A/B-II in cells exposed to UA/ZOL combination was markedly enhanced, suggesting an additive effect between UA and ZOL in both lines of osteosarcoma cells (Figure 5A). We also confirmed an enhanced formation of autophagic vacuoles as appeared as red fluorescent in cells exposed to both UA and ZOL, compared to those treated with a single compound (Figure 5B). Given that autophagy can either enhance cell survival or be cytotoxic, cell viability was determined using MTT assays to evaluate the role of UA/ZOL-induced autophagy in osteosarcoma cells. We showed that UA/ZOL combination has greatly reduced cell viability compared to control group (Figure 5C). Interestingly, in the presence of autophagy antagonist 3-methyladenine (3-MA), the UA/ZOL combination failed to suppress cell viability, indicating that the combined anti-proliferative effect was rescued by the autophagy inhibitor (Figure 5C). These various lines of evidence all support that autophagy contributes to the combined anti-growth effect of ZOL and UA in osteosarcoma cells.

### 2.2. ROS Was Associated with Apoptosis Induced by Combination of Zoledronic Acid and Ursolic Acid

**Figure 6:**
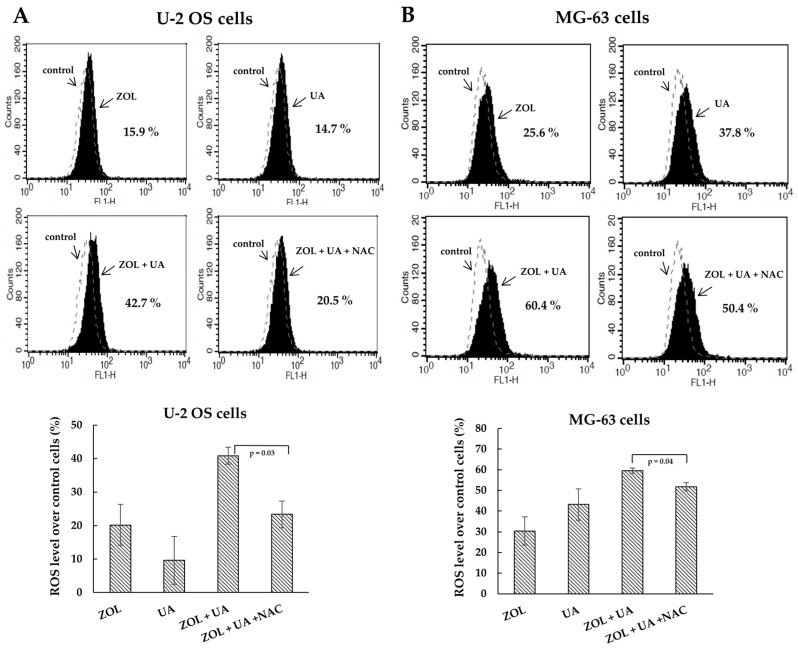
Zoledronic acid combines with ursolic acid to enhance reactive oxygen species (ROS) generation. U-2 OS cells (**A**) or MG-63 cells; (**B**) were treated with either ZOL (10 μM), UA (20 μM) or a combination of ZOL/UA in the absence or presence of *N*-acetyl-l-cysteine (NAC) (2 μM) for 16 h. The cells were stained with hydroethidine (10 μM) and ROS levels were analyzed by flow cytometry. Numbers indicated the induced levels of ROS in drug-treated cells compared to control cells. The results were calculated by a median of fluorescence intensity in each group.

**Figure 7:**
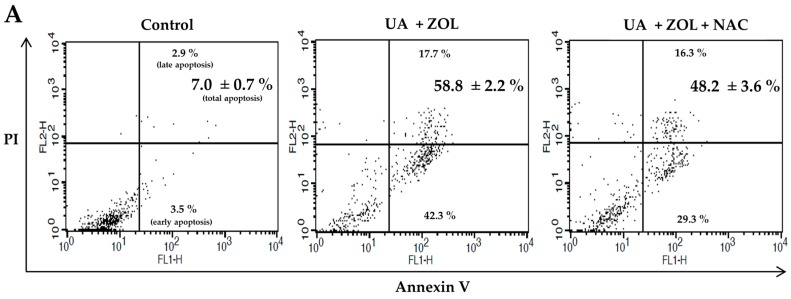

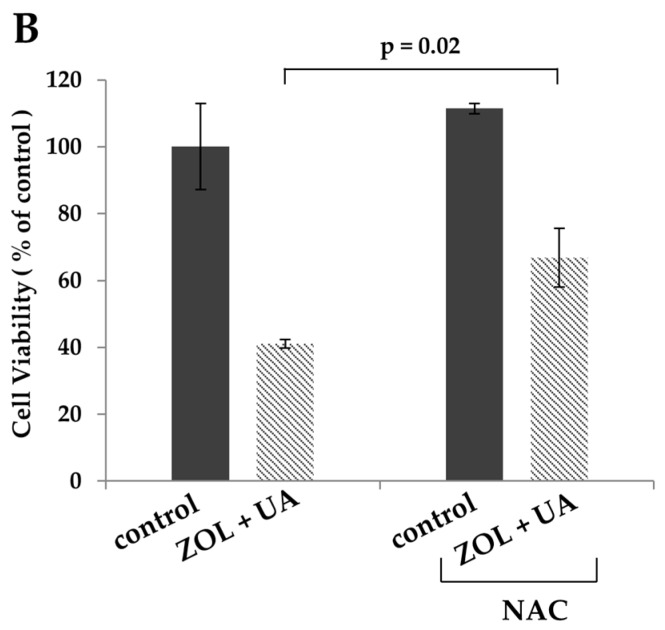
ROS is involved in the apoptosis effect of the UA/ZOL combination. (**A**) ROS inhibitor, *N*-acetyl-l-cysteine (NAC) decreases the apoptosis induced by the combination of ZOL and UA. U-2 OS cells were treated with ZOL (10 μM) and UA (20 μM) in the absence or presence of NAC (2 μM) for 48 h. The treated cells were stained with Annexin-V and PI to analyze the percentage of apoptosis cells as indicated numbers by flow cytometry; and (**B**) NAC rescues the cell viability inhibition caused by the combination of ZOL and UA determined by MTT assays.

Reactive oxygen species (ROS) are shown to be involved in cell death induced by many anticancer drugs [26]. To address the mechanism of the combined anti-proliferative effect of UA/ZOL, ROS generation was evaluated. We utilized fluorescent probe hydroethidine to determine the level of intracellular ROS in cells exposed to either a single compound or UA/ZOL combination. As shown in Figure 6, the UA/ZOL combination increased the intracellular ROS level as compared with a single compound in U-2 OS cells (Figure 6A). Similar results were also observed in MG-63 cells (Figure 6B). We also examined the effect of ROS scavenger *N*-acetyl-l-cysteine (NAC) in this system and found that NAC significantly suppressed the ROS generation induced by UA/ZOL combination (Figure 6). The NAC-attenuated UA/ZOL-induced ROS generation was also paralleled by a significant reduction of apoptosis (Figure 7A). As a result, this combined anti-growth effect of UA/ZOL was partially rescued by NAC treatment (Figure 7B), supporting an essential role of ROS in the combined effect of UA/ZOL in osteosarcoma cells.

## 3. Discussion

In this report, we demonstrated that ZOL combined with UA to enhance its anti-growth effect in cell viability and colony formation assays in two lines of human osteosarcoma cells. The co-treatment with ROS scavenger or autophagy inhibitor partially reversed the combined anti-growth effect of UA/ZOL, suggesting oxidative stress and autophagy are both important in the combined cytotoxic effect of UA and ZOL.

ZOL has been widely applied in the treatment of osteoporosis. Recent reports showed that ZOL also has the anti-cancer effect in many different tumors by inhibition of cancer cell proliferation or tumor metastasis [10,11,17]. The recent focus is on enhancing the anti-cancer activity of ZOL by the combination of other anticancer drugs. This approach has resulted in improved responses and the ability to use lower and less toxic concentrations of the drugs. One such example is that cisplatin has the synergistic effect with ZOL to inhibit proliferation of breast and lung cancer cells [27,28]. Moreover, the combined effects of ZOL and ionizing radiation have been evaluated in human fibrosarcoma cells, in the hope of providing a potential therapy for patients with soft tissue sarcoma [29].

Ursolic acid (UA), a naturally occurring pentacyclic triterpene acid, is the major component of certain traditional medicine herbs and edible plants. It is demonstrated to be apoptotic, resulting in the suppression of cell growth in various human cancers [30,31]. Most recently, we have validated that UA triggers marked apoptosis in human osteosarcoma cells, whereas little apoptosis is induced in non-cancerous cells [9]. Thus, it is of interest to study the combined effect between UA and ZOL. Our data confirmed that the combined treatment with UA and ZOL largely suppressed the proliferation of osteosarcoma cells, a degree that is stronger than the combined effect of CPT and ZOL (Figure 2). The results of the combination of ZOL and UA are interesting and novel, providing a foundation for future clinical application in the treatment of the patients with osteosarcoma.

One major finding of this study is our elucidation of the mechanism underlying the combination of UA and ZOL on anti-proliferation of osteosarcoma cells. We clearly demonstrate that ROS generation and autophagy are important for this combined effect of UA/ZOL. Autophagy is a cellular process used to degrade and turn over proteins and cytoplasmic organelles in response to stresses. However, the cellular outcome for autophagy induction is multifaceted and depends on the context of cells. It is suggested that autophagy exhibits dual functions to suppress and enhance cell growth [32,33,34]. ZOL is shown to induce autophagy in different lines of cancers including prostate, cervical, and in salivary adenoid cystic carcinoma. In these studies, the inhibition of autophagy decreases ZOL-induced cell death, suggesting that ZOL-induced autophagy is cytotoxic [35,36,37]. Consistent with others, we also observed that treatment with autophagy inhibitor has reversed UA/ZOL-induced autophagy, and concurrently enhanced cell viability in U-2 OS cells. Thus, UA/ZOL combination increased autophagy induction, leading to enhanced apoptosis and reduced cell viability and cell proliferation (Figure 5).

Accumulating data have suggested that ROS signaling functions as the mediator of cell growth that either stimulate proliferation or induce cell death [38]. Previous studies have described that ZOL or UA treatment induce ROS-dependent apoptosis in different cancer cells [9,39,40]. Moreover, other groups have shown that ROS generation contributes to the significantly enhanced apoptosis by the combination of ZOL and ionizing radiation or panobinostat [41,42]. Similarly, in this study, we found that either ZOL or UA triggered ROS elevation accompanied by apoptosis induction, and the UA/ZOL combination considerably enhanced these responses. Importantly, ROS scavenger NAC partially suppressed the apoptosis induced by the UA/ZOL combination, indicating that that ROS generation is associated with the combined effect of UA/ZOL in apoptosis induction in osteosarcoma cells (Figure 7).

## 4. Materials and Methods 

### 4.1. Cell Culture and Reagents

Two lines of human osteosarcoma cells were used in this study. U-2 OS cells were obtained from Dr. Sheau-Yann Shieh (Institute of Biomedical Sciences, Sinica Academia, Taipei, Taiwan) and MG-63 cells were purchased from the Bioresource Collection and Research Center (Hsinchu, Taiwan). Both lines of cells were maintained in Dulbecco’s modified Eagle’s medium (DMEM) supplemented with 10% fetal bovine serum (Gibco, Carlsbad, CA, USA), 100 U/mL of penicillin, and 100 g/mL of streptomycin (Gibco). Zoledronic acid and cisplatin were obtained from Sigma Chemical Co. (St. Louis, MO, USA). Ursolic acid, *N*-acetyl-l-cysteine, acridine orange, and 3-methyladenine were obtained from Santa Cruz Biotechnology (Santa Cruz, CA, USA).

### 4.2. Cell Lysis and Immunoblotting

Cells were lysed in TEGN buffer (10 mM Tris, pH 7.5, 1 mM EDTA, 420 mM NaCl, 10% glycerol, and 0.5% Nonidet P-40) containing proteases inhibitor cocktail (Roche) and 1 mM dithiothreitol (DTT). For Western blotting, the cell lysates were boiled in protein sample buffer (2 M β-mercaptoethanol, 12% sodium dodecyl sulfate (SDS), 0.5 M Tris, pH 6.8, 0.5 mg/mL bromophenol blue, and 30% glycerol), and analyzed by SDS-polyacrylamide gel electrophoresis (PAGE). Antibodies used were the following: LC3A/B antibody (#4108; Cell Signaling, Beverly, MA, USA), actin (A2066; Sigma), cleaved caspase-3 (#9661; Cell Signaling), PARP (#9542; Cell Signaling).

### 4.3. MTT Assay

For measuring cell viability, the MTT [3-(4,5-dimethylthiazol-2-y1)-2,5-diphenyltetrazolium bromide] assay was performed. 2 × 10^3^ cells per well were seeded in 96-well plates and cultured for different times. At the end of the assay time, 10 μL of MTT solution (5 mg/mL) (Invitrogen, Carlsbad, CA, USA) was added to each well, and then incubated for 4 h at 37 °C. After removing the cultured medium, 200 μL of dimethyl sulfoxide (DMSO) was added to each well, and the plates were read at 540 nm using a spectrophotometric plate reader with a reference wavelength at 650 nm.

### 4.4. Colony Formation Assay

MG-63 cells were treated with ursolic acid (UA) (20 μM), zoledronic acid (ZOL) (10 μM), or co-treated with UA and ZOL for 6 h. Cells after exposure were trypsinized, plated and maintained onto 35 mm dishes (500 cells/dish) for 10 to 14 days to allow colony formation. Colonies were fixed in 70% ethanol and stained by 1% crystal violet solution before counting.

### 4.5. Measurement of Oxidative Stress (ROS) Determination

Level of intracellular ROS were using ROS-sensitive dye hydroethidine (Santa Cruz). The cells were trypsinized, washed one time with phosphate-buffered saline (PBS) and incubated with hydroethidine (10 μM) in 400 μL of DMEM medium with 10% fetal bovine serum for 30 min at 37 °C. Finally, the cells were washed two times by PBS, and the fluorescence was detected by a Cytomics™ FC500 flow cytometer (Beckman Coulter, Miami, FL, USA). A minimum of 20,000 cells were collected and analyzed to determine median of fluorescence intensity in each group.

### 4.6. Apoptosis Assay

Cell apoptosis was analyzed by flow cytometry using the Annexin-V-FITC staining kit (Becton Dickinson, San Jose, CA, USA) according to the manufacturer’s instructions. Briefly, the cells were trypsinized, and washed twice by cold PBS. The cells were incubated with 100 μL of 1× binding buffer with 5 μL of FITC Annexin V and 5 μL of propidium iodide (PI) for 15 min at room temperature (RT) in the dark. After incubation, 400 μL of 1× binding buffer was added to each tube, and the fluorescence was detected by a Cytomics™ FC500 flow cytometer (Beckman Coulter, Miami, FL, USA).

### 4.7. Autophagy Determination

Autophagosomes were visualized by staining with Acridine Orange (AO). After incubation, cells were washed with PBS and stained with AO (1 μM) for 10 min at 37 °C. AO-stained cells were washed, counter-stained with 4′,6-diamidino-2-phenylindole (DAPI), and examined by an Olympus IX81 microscope (Olympus, Tokyo, Japan). The stained lysosomes or acidic vacuoles were observed as red foci in the cells.

### 4.8. Statistical Analysis

All data are expressed as the mean ± SD of no less than three independent trials. The differences between groups were calculated using a Student’s *t*-test provided by the GraphPad Prism (Version 4.0, GraphPad Software; San Diego, CA, USA).

## 5. Conclusions

In conclusion, our results indicate that ZOL combines with UA to attenuate cell viability and colony formation in two lines of osteosarcoma cells, and ROS generation and autophagy are important in these processes. The findings of this study assist us in understanding the combined anti-proliferative effect of UA/ZOL, and hope to provide a rational framework for the further development of improved strategies in the treatment of patients with osteosarcoma.
